# Comparison adductor canal block combined with local infiltration analgesia and adductor canal block alone for pain management after total knee arthroplasty: A randomized controlled trial protocol

**DOI:** 10.1097/MD.0000000000021881

**Published:** 2020-08-28

**Authors:** Qingchun Zhang, Limei Fan

**Affiliations:** Department of Anesthesiology, Jinxiang Hospital Affiliated to Jining Medical College, Shandong, China.

**Keywords:** adductor canal block, local infiltration analgesia, pain, protocol, total knee arthroplasty

## Abstract

**Background:**

Pain control after total knee arthroplasty has shown many advances; however, the optimal method remains controversial. The purpose of this present study is to assess the efficacy and safety of the addition of local infiltration analgesia to adductor canal block for pain control after primary total knee arthroplasty.

**Methods:**

This prospective randomized controlled research was conducted from January 2018 to June 2019. All the patients and their family members signed the informed consent forms, and this work was authorized via the ethics committee of Jinxiang Hospital Affiliated to Jining Medical College (JXHP0024578). Inclusion criteria were 55 years old or older, who possess the physical status I–III of American Society of Anesthesiologists, and the body mass index in the range of 18 to 30 kg/m^2^. Exclusion criteria were regional and/or neuroaxial anesthesia contraindications, the history of drug allergy involved in the research, neuropathic pain, as well as the chronic pain requiring opioid therapy. Seventy-two patients were divided into 2 groups randomly. Study group (n = 36) received both adductor canal block and local infiltration analgesia. Control group (n = 36) received adductor canal block alone. Primary outcome included postoperative pain score (visual analog scale 0 to 10 cm, in which 0 represents no pain and 10 represents the most severe imaginable pain). The measures of secondary outcome included the knee range of motion, opioid consumption, the hospital stay length as well as the postoperative complications (for instance, pulmonary embolism, deep vein thrombosis, and the wound infection). All the analyses were conducted through utilizing the SPSS for Windows Version 20.0.

**Results:**

The results will be shown in Table 1

**Table 1:**
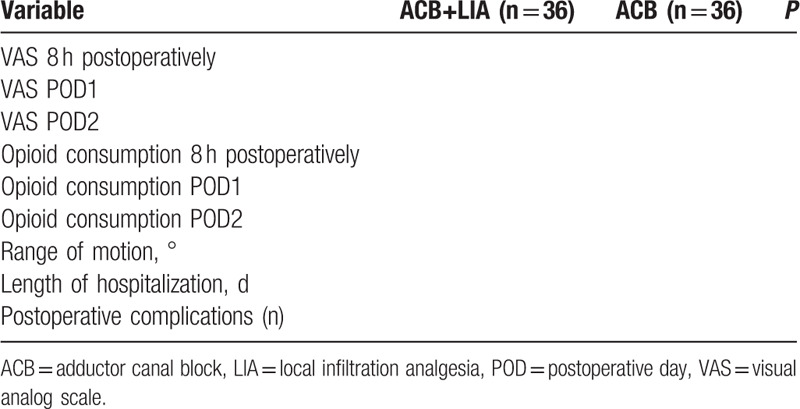
Comparison of outcomes between groups.

.

**Conclusion:**

The study will provide more evidence on the combination use of adductor canal block and local infiltration analgesia in the treatment of pain after the total knee arthroplasty.

**Trial registration:**

This study protocol was registered in Research Registry (researchregistry5832).

## 1 Introduction

Total knee arthroplasty (TKA) is commonly conducted to address the pain and functional disorder, which attends end-stage osteoarthritis and rheumatoid arthritis.^[1,2]^ In the wake of the aging of the U.S. population, the number of knee arthroplasty is expected to obviously increase by 2030, with the estimated 3.48 million cases each year.^[3]^ However, postoperative pain remains a major complication, and pain control is an essential component of optimal care in surgical patients. Failure to provide adequate analgesia may affect physical rehabilitation, which is important to improve joint range of motion and promote satisfactory results.^[4]^ An extended period of postoperative inactivity may potentially increase medical costs, as well as aggravating the risk of thromboembolism, such as deep venous thrombosis and pulmonary embolism.^[5,6]^

Several techniques have been introduced for the management of postoperative pain, including the epidural analgesia, patient-controlled intravenous analgesia, femoral nerve block, and multimodal cocktail periarticular injection.^[7–10]^ In comparison with the femoral nerve block, ultrasound-guided adductor canal block (ACB) can improve the strength of quadriceps femoris and is extensively utilized in the pain control after TKA. However, isolated ACB fails to provide adequate analgesia to the posterior knee. The duration of local infiltration analgesia (LIA) is short, which limits its clinical application.^[11]^ Barastegui et al^[12]^ reported that periarticular infiltration analgesia was effective and safe to decrease the perioperative pain 36 hours after TKA. Its effects will disappear with the passage of time, but it will not change the postoperative course of treatment, and will not affect the satisfaction of patients with short-term follow-up. Recent published studies have indicated that ACB in combination with LIA may achieve satisfactory effects, as well as an improved functional outcome.^[13,14]^

Currently, whether ACB combined with LIA is superior to isolated ACB remains controversial, due to the small number of the published articles examining the efficacy of each modality. The purpose of this present study is to assess the efficacy and safety of the addition of LIA to ACB for pain control after primary TKA. We hypothesize that ACB combined with LIA will provide better pain relief and in the immediate postoperative period compared with ACB alone.

## 2 Materials and methods

### 2.1 Enrollment

This prospective randomized controlled research was conducted from January 2018 to June 2019 and was carried out on the basis of SPIRIT Checklist for randomized studies. It was authorized via the Institutional Review Committee in Jinxiang Hospital Affiliated to Jining Medical College (JXHP0024578) and then was registered in research registry (researchregistry5832). Each patient received the written informed consent. Patients scheduled for a primary TKA were identified at the preoperative evaluation clinic, and in a private office, they were invited to meet with a research assistant who would confirm the eligibility, and interpret the research, and acquire the informed written consent. Inclusion criteria were 55 years or older, who possess the physical status I–III of American Society of Anesthesiologists, and the body mass index in the range of 18 to 30 kg/m^2^. Exclusion criteria were regional and/or neuroaxial anesthesia contraindications, the history of drug allergy involved in the research, neuropathic pain, as well as the chronic pain requiring opioid therapy.

### 2.2 Study design

Seventy-two patients were divided into 2 groups randomly through utilizing the computer-generated forms and the drawing-coded sealed opaque envelopes. The anesthesiologist who conducted the block knew the treatment, but both the research assistant and the patient did not know about the grouping assignment. Study group (n = 36) received both ACB and LIA. Control group (n = 36) received ACB alone.

### 2.3 Surgical procedure

All patients were given the general anesthesia. The surgical procedures were performed by the senior surgeon. In the process of operation, pneumatic tourniquet was utilized. An incision was made in the center of the knee and then extended to the medial side of patella. All the ACB operations were conducted via an experienced anesthesiologist under the guidance of ultrasound. The patients were kept in the supine site, with the knee joint slightly bent and the leg rotated externally, and then 0.5% of the chlorhexidine spray skin was prepared. The ultrasound probe was utilized for the identification of the femoral artery and sartorius muscle. By passing through the sartorius into the adductor muscle duct and placing the needle appropriately until the needle tip could be seen by ultrasound. The needle aspiration was carried out to ensure that the femoral artery was not penetrated, and the experimental dose of 1 mL local anesthetic was injected. Ultrasound was used to observe whether the local anesthetic was diffused in internal adductor tube, so as to confirm the correct placement of the needle. Five to 10 milliliters 0.2% to 0.75% ropivacaine or 0.25% to 0.5% bupivacaine were then injected into adductor tube. The LIA was implemented utilizing 150 mg of ropivacaine, 30 mg of ketorolac, and adrenaline 200 μg as well as 10 mg of morphine, with 75 mL total volume, administered intraoperatively via surgeon.

### 2.4 Outcome measures

Preoperative and postoperative clinical data were evaluated by an independent senior surgeon blinded to the patient's randomization. Primary outcome included postoperative pain score (visual analog scale 0–10 cm, in which 0 represents no pain and 10 represents the most severe imaginable pain). Pain assessment was performed 12 hours after surgery, and pain was recorded at rest on the first and second days after surgery. The measures of secondary outcome included the knee range of motion, opioid consumption, the hospital stay length as well as the postoperative complications (for instance, pulmonary embolism, deep vein thrombosis, and the wound infection).

### 2.5 Statistical analysis

The calculations of sample size was conducted on the basis of our preliminary study. The results showed that ACB combined with LIA and ACB only reduced 24-hour postoperative opioid consumption from 14.9 ± 3.7 to 12.1 ± 4.8 mg. The number of 32 participants per group will provide 80% power to detect the equal difference in 24-hour opioid consumption at 2-sided alpha of 0.05. The definite 4 number of participants per group was 36 after compensation of data loss. All the analyses were implemented through utilizing SPSS for Windows Version 20.0. All the data are represented with proper characteristics as median, mean, percentage as well as standard deviation. Mann–Whitney *U* test or the independent samples *t* test were used to analyze the inter group comparison. Chi-square detection was utilized to compare the categorical variables among the groups. Repeated data were analyzed by repeated measured analysis of variance. The *P* < .05 was regarded the significant in statistics.

## 3 Result

The results are summarized in Table 1.

## 4 Discussion

As the population ages, knee osteoarthritis has become more and more common. TKA has been widely performed for patients aged 60 years or older and it has become a serious public health issue. Meanwhile, approximately half of the patients undergoing TKA suffer from moderate to severe postoperative pain and effective pain control is regarded as a key to obtaining early ambulation and rehabilitative. Currently, there is still no reliable evidence or extensively accepted guideline for the best postoperative analgesia. Expert consensus has recommended the application of multimodal analgesia for reducing pain and opioid consumption after lower extremity surgery.^[15]^ The adductor canal consists of the medial femoral cutaneous nerve, medial femoral nerve, the posterior articular branches of obturator nerve, and sometimes the anterior branch from obturator nerve.^[16]^ Previous studies have reported that ACB showed similar pain relief and superior strength of musculi quadriceps femoris in comparison with femoral nerve block, and could thereby decrease the risk of falls during the postoperative rehabilitation process.^[17,18]^ However, isolated ACB cannot provide complete analgesia to the posterior knee and LIA has a short-term action leading to less than satisfactory pain relief. Our research compares the efficacy of ACB combined with LIA and ACB alone in the treatment of postoperative pain after TKA. The sample size of our study is relatively small, and results may be underpowered to evaluate the efficacy of ACB along with LIA. High quality of randomized controlled trials with large sample size is required to convince our results.

## 5 Conclusion

The study will provide more evidence on the combination use of ACB and LIA in the treatment of pain after the TKA.

## Author contributions

Limei Fan planned the study design and reviewed the study protocol. Qingchun Zhang has written the protocol and will recruit participants and collect data. All of them have read, commented on, and contributed to the submitted manuscript.

**Writing – original draft:** Qingchun Zhang.
